# A common variant of leucine-rich repeat-containing 16A (*LRRC16A*) gene is associated with gout susceptibility

**DOI:** 10.1007/s13577-013-0081-8

**Published:** 2013-12-07

**Authors:** Masayuki Sakiyama, Hirotaka Matsuo, Seiko Shimizu, Toshinori Chiba, Akiyoshi Nakayama, Yuzo Takada, Takahiro Nakamura, Tappei Takada, Emi Morita, Mariko Naito, Kenji Wakai, Hiroki Inoue, Seishiro Tatsukawa, Junki Sato, Kazumi Shimono, Toshiaki Makino, Takahiro Satoh, Hiroshi Suzuki, Yoshikatsu Kanai, Nobuyuki Hamajima, Yutaka Sakurai, Kimiyoshi Ichida, Toru Shimizu, Nariyoshi Shinomiya

**Affiliations:** 1 Department of Integrative Physiology and Bio-Nano Medicine, National Defense Medical College, 3-2 Namiki, Tokorozawa, Saitama 359-8513 Japan; 2 Department of Dermatology, National Defense Medical College, Tokorozawa, Japan; 3 Laboratory for Biofunctions, The Central Research Institute, National Defense Medical College, Tokorozawa, Japan; 4 Laboratory for Mathematics, National Defense Medical College, Tokorozawa, Japan; 5 Department of Pharmacy, The University of Tokyo Hospital, Faculty of Medicine, The University of Tokyo, Tokyo, Japan; 6 Department of Preventive Medicine, Nagoya University Graduate School of Medicine, Nagoya, Japan; 7 Department of Pharmacokinetics, Faculty of Pharmaceutical Sciences, Toho University, Funabashi, Japan; 8 Department of Pharmacognosy, Graduate School of Pharmaceutical Sciences, Nagoya City University, Nagoya, Japan; 9 Division of Bio-system Pharmacology, Department of Pharmacology, Osaka University, Osaka, Japan; 10 Department of Healthcare Administration, Nagoya University Graduate School of Medicine, Nagoya, Japan; 11 Department of Preventive Medicine and Public Health, National Defense Medical College, Tokorozawa, Japan; 12 Department of Pathophysiology, Tokyo University of Pharmacy and Life Sciences, Tokyo, Japan; 13 Midorigaoka Hospital, Takatsuki, Japan

**Keywords:** Gouty arthritis, Single nucleotide polymorphism (SNP), Urate transport, PDZ domain-containing 1 (PDZK1), Sodium–proton exchanger regulatory factor 1 (NHERF1)

## Abstract

Gout is a common disease resulting from hyperuricemia which causes acute arthritis. Recently, genome-wide association studies revealed an association between serum uric acid levels and a common variant of leucine-rich repeat-containing 16A (*LRRC16A*) gene. However, it remains to be clarified whether *LRRC16A* contributes to the susceptibility to gout. In this study, we investigated the relationship between rs742132 in *LRRC16A* and gout. A total of 545 Japanese male gout cases and 1,115 male individuals as a control group were genotyped. rs742132 A/A genotype significantly increased the risk of gout, conferring an odds ratio of 1.30 (95 % CI 1.05–1.60; *p* = 0.015). *LRRC16A* encodes a protein called capping protein ARP2/3 and myosin-I linker (CARMIL), which serves as an inhibitor of the actin capping protein (CP). CP is an essential element of the actin cytoskeleton, which binds to the barbed end of the actin filament and regulates its polymerization. In the apical membrane of proximal tubular cells in the human kidney, the urate-transporting multimolecular complex (urate transportsome) is proposed to consist of several urate transporters and scaffolding proteins, which interact with the actin cytoskeleton. Thus, if there is a CARMIL dysfunction and regulatory disability in actin polymerization, urate transportsome may be unable to operate appropriately. We have shown for the first time that *CARMIL/LRRC16A* was associated with gout, which could be due to urate transportsome failure.

**Electronic supplementary material:**

The online version of this article (doi:10.1007/s13577-013-0081-8) contains supplementary material, which is available to authorized users.

## Introduction

The leucine-rich repeat-containing 16A (*LRRC16A*) gene encodes a protein called capping protein ARP2/3 and myosin-I linker (CARMIL), which plays an important role in cell-shape changes and motility [1]. A common variant of *LRRC16A* gene has been previously reported to be associated with nephrolithiasis [2], platelet count [3], and hemoglobin [4]. In addition, a meta-analysis of genome-wide association studies (GWAS) has revealed an association between serum uric acid (SUA) levels and rs742132, a single nucleotide polymorphism (SNP) in *LRRC16A* [5]. While elevated SUA levels potentially cause gout [6], it remains to be clarified whether *LRRC16A* contributes to the susceptibility to gout. In this study, therefore, we investigated the effects of a common variant of *LRRC16A* on the susceptibility to gout.

## Materials and methods

### Study participants

All procedures were carried out in accordance with the standards of the institutional ethical committees involved in this project and the Declaration of Helsinki with written informed consent from each subject participating in this study. For cases, 545 male gout patients were assigned from among outpatients of gout clinics in Midorigaoka Hospital (Osaka, Japan). All were clinically diagnosed with primary gout according to the criteria established by the American College of Rheumatology [7]. For the control group, 1,115 males with normal SUA (≤7.0 mg/dl) and without a history of gout were collected from the Japan Multi-Institutional Collaborative Cohort Study (J-MICC Study) [8]. The details and participants in this study are shown in Supplemental Table 1.

### Genetic analysis

Genomic DNA was extracted from whole peripheral blood cells [9]. Genotyping of rs742132, a common variant of *LRRC16A* gene, was performed by TaqMan method (Life Technologies, Carlsbad, CA, USA) with a LightCycler 480 (Roche Diagnostics, Mannheim, Germany) [10, 11]. To confirm their genotypes, DNA sequencing analysis was performed with the following primers: forward 5′-GATCACACTGTGACCACACC-3′, and reverse 5′-GTATCTCTGTGCCTCATATTCCTC-3′. Direct sequencing was performed with a 3130xl Genetic Analyzer (Life Technologies) [11].

For all calculations in the statistical analysis, SPSS v.17.0J (IBM Japan, Tokyo, Japan) were used. The Chi-square test was used for association analysis.

## Results

Genotyping results of rs742132 for 545 gout patients and 1,115 controls are shown in Table 1. The call rate for rs742132 was 97.3 %. Its *p* value for Hardy–Weinberg equilibrium was 0.56 in controls. A *p* value that suggested mistyping was not obtained. The association analysis (2 × 3 Chi-square test) of the *LRRC16A* variant, rs742132, showed a significant result (*p* = 0.027; Table 1). The A-allele of rs742132 was a risk allele for gout in this study, and the risk allele frequency in the gout cases (75.0 %) was higher than in the controls (72.0 %; Table 1). As a result, rs742132 had a borderline significant association for the allele frequency model (*p* = 0.070; Table 2). In addition, the A/A genotype was observed more frequently in the gout cases (58.5 %) than in the control subjects (52.1 %; Table 1). Although no significant association was observed in the dominant model (*p* = 0.784), A/A genotype significantly increased gout risk in the recessive model (*p* = 0.015; odds ratio = 1.30; 95 % CI 1.05–1.60; Table 2).

**Table 1:** Distributions of genotypes of rs742132 in *LRRC16A* gene

	G/G	G/A	A/A	RAF^a^	*p* value^b^
Gout cases	47	179	319	0.750	0.027
Controls	88	424	558	0.720	–

*RAF* Risk allele frequency

^a^A is risk allele

^b^2 × 3 Chi square test of rs742132 genotype

**Table 2:** The risk of gout due to a common variant of *LRRC16A* gene, rs742132

	*p* value	OR	95 % CI
Allele frequency model	0.070	1.17	0.99–1.38
Recessive model (G/G or G/A versus A/A)	0.015	1.30	1.05–1.60
Dominant model (G/G versus G/A or A/A)	0.784	0.95	0.66–1.37

*OR* odds ratio, *CI* confidence interval

## Discussion

Gout is a common disease as a consequence of hyperuricemia which increases the risks of hypertension [6, 12], cardiovascular diseases [13], cerebrovascular diseases [14], and renal failure [15]. Previous studies identified several transporter genes associated with gout, such as ATP-binding cassette transporter, subfamily G, member 2 (*ABCG2/BCRP*) [11, 16–18], *GLUT9/SLC2A9* [19–21], monocarboxylate transporter 9 (*MCT9/SLC16A9*) [22], and organic anion transporter 4 (*OAT4/SLC22A11*) [23]. In the present study, we have shown for the first time that a common variant of *LRRC16A* has a significant association with gout. Although rs742132 is reported to associate with SUA [5], another study revealed no significant association between *LRRC16A* and gout [19]. This is partly because the participants in that study were medical history reading or self-reported patients, whereas we performed this study using only clinically diagnosed cases for a better understanding of the genetic basis of gout. While the functional role of rs742132 remains unknown and further studies are necessary, it may well be possible that this intronic SNP would regulate *LRRC16A* gene expression or be a surrogate marker for other functional SNPs.

*LRRC16A* encodes CARMIL, a large protein which is the most abundant in kidney and other epithelial tissues [1]. It serves as an inhibitor of the heterodimeric actin capping protein (CP), an essential element of the actin cytoskeleton which binds to the barbed end of the actin filament and regulates its polymerization [1, 24] (Supplemental Fig. 1). Therefore, *LRRC16A* mutation may cause the dysfunction of CARMIL to dislodge the capping protein from the actin filament which results in uncontrolled elongation at the barbed end of the filament.

**Fig. 1:**
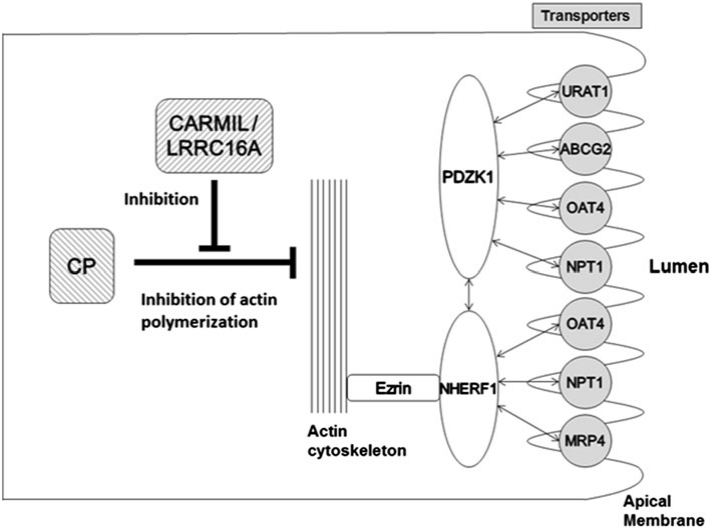
A proposed model of CARMIL/LRRC16A-mediated urate transportsome regulation. In the urate transportsome of renal proximal tubular cells, urate transporters are scaffolded by PDZK1 and NHERF1, which interacted with the actin cytoskeleton through ezrin [ref. 25, 26]. In this study, we propose a new model of urate transportsome regulation by CARMIL. In this model, CARMIL dysfunction, which causes uncontrolled elongation of actin filament, could relate to the pathophysiology of gout

Recently, in the apical membrane of proximal tubular cells in the human kidney, a urate-transporting multimolecular complex (urate transportsome) [25] is proposed to be composed of the following transporters: urate transporter 1 (URAT1/SLC22A12), ABCG2/BCRP, OAT4/SLC22A11, type 1 sodium-dependent phosphate transporter (NPT1/SLC17A1), and multidrug resistance protein 4 (MRP4/ABCC4) [26] (Fig. 1). These transporters are scaffolded by a PDZ domain-containing 1 (PDZK1) and sodium–proton exchanger regulatory factor 1 (NHERF1) [26]. NHERF1 interacts with the actin cytoskeleton through the ezrin protein. Hence, if there is CARMIL dysfunction and regulatory disability in actin polymerization, urate transportsome may be unable to operate appropriately, which results in urate transport failure (Fig. 1). In addition to these transporters, shown in Fig. 1, a type 4 sodium-dependent phosphate transporter (NPT4/SLC17A3) is also reported to be a urate transporter expressed in kidney [27]. CARMIL may also have effects on NPT4 by regulating urate transportsome, because NPT4 is supposed to bind PDZK1 and/or NHERF1.

Until now, the multiple biochemical mechanisms associated with CARMIL raise many possibilities for its intracellular function [1, 24]. We suggest that CARMIL/LRRC16A has a novel mechanism associated with gout due to urate transportsome failure.

## Electronic supplementary material

Below is the link to the electronic supplementary material.

Supplementary material 1 (PDF 7 kb)

Supplementary material 2 (TIFF 105 kb)
