# Supplementary material for: A common variant of leucine-rich repeat-containing 16A (LRRC16A) gene is associated with gout susceptibility
Source: Hum Cell. 2013 Dec 7;27(1):1–4. doi: 10.1007/s13577-013-0081-8 (PMC3889988; doi:10.1007/s13577-013-0081-8)
Supplement: Supplementary file 1 — Supplementary material 1 (PDF 7 kb) [file 13577_2013_81_MOESM1_ESM.pdf]

Supplemental Table 1 Clinical characteristics of the participants

	Case			Control		
Number	545			1,115		
Age (year)	54.2	±	13.4	52.6	±	8.3
Body-mass index (kg/m^2)	24.8	±	3.6	23.2	±	2.8

Plus-minus values are means ± SD
