# Supplementary figures and images for: A common variant of leucine-rich repeat-containing 16A (LRRC16A) gene is associated with gout susceptibility
Source: Hum Cell. 2013 Dec 7;27(1):1–4. doi: 10.1007/s13577-013-0081-8 (PMC3889988; doi:10.1007/s13577-013-0081-8)

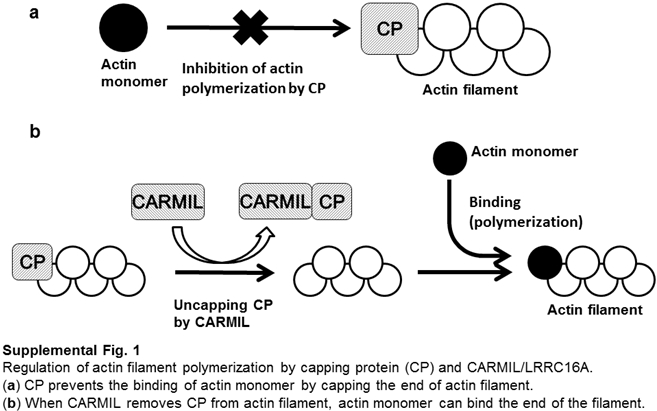

Supplement: Supplementary file 2 — Supplementary material 2 (TIFF 105 kb) [file 13577_2013_81_MOESM2_ESM.tif]
